# 

**Published:** 1889-03-02

**Authors:** 


PERMANENTLY ENLARGED TO 32 PAGES.

PRICE ONE PENNY.

No. 127, Vol. V.—March 2nd, 1889.

[Registered at the General Post Office as a Newspaper.]

Per Annum, 6s. 6d., post free.
Monthly Parts, 6d.; post free, 7½d.
Ditto per Ann. 7s. 6d., post free.

